# Unsupervised Monocular Depth Estimation for Colonoscope System Using Feedback Network

**DOI:** 10.3390/s21082691

**Published:** 2021-04-11

**Authors:** Seung-Jun Hwang, Sung-Jun Park, Gyu-Min Kim, Joong-Hwan Baek

**Affiliations:** School of Electronics and Information Engineering, Korea Aerospace University, Goyang 10540, Korea; fogfog2@kau.kr (S.-J.H.); tjdwns1011@naver.com (S.-J.P.); gyumin46@naver.com (G.-M.K.)

**Keywords:** unsupervised deep learning, monocular depth estimation, colonoscopy

## Abstract

A colonoscopy is a medical examination used to check disease or abnormalities in the large intestine. If necessary, polyps or adenomas would be removed through the scope during a colonoscopy. Colorectal cancer can be prevented through this. However, the polyp detection rate differs depending on the condition and skill level of the endoscopist. Even some endoscopists have a 90% chance of missing an adenoma. Artificial intelligence and robot technologies for colonoscopy are being studied to compensate for these problems. In this study, we propose a self-supervised monocular depth estimation using spatiotemporal consistency in the colon environment. It is our contribution to propose a loss function for reconstruction errors between adjacent predicted depths and a depth feedback network that uses predicted depth information of the previous frame to predict the depth of the next frame. We performed quantitative and qualitative evaluation of our approach, and the proposed FBNet (depth FeedBack Network) outperformed state-of-the-art results for unsupervised depth estimation on the UCL datasets.

## 1. Introduction

According to Global Cancer Statistics 2018 [1], colorectal cancer causes approximately 90,000 deaths worldwide each year, with the highest incidence rates in Europe, Australia, New Zealand, North America, and Asia. Colonoscopy is a test for the detection and removal of polyps, and it can prevent cancer by detecting adenoma. However, the polyp detection rate varies according to the condition and skill level of the endoscopist, and even some endoscopists have a 90% chance of missing an adenoma [2]. Endoscopy doctors’ fatigue and skill problems can be compensated for by artificial intelligence and robotic medical systems [3]. Recently, polyp detection [4], size classification [5], and detecting deficient coverage in colonoscopy [6] have been proposed as computer-assisted technologies using artificial intelligence. In the field of robotic colonoscopy technology, there are studies on conventional colonoscope miniaturizing [3], robotic meshworm [7], treaded capsule [8], and autonomous locomotion system [9] to facilitate colonoscopy.

In general, computer-assisted endoscopic imaging systems are mainly studied based on the monocular camera because it is difficult to utilize a stereo camera according to the size limitation of each organ [10,11] Monocular depth estimation, which provides spatial information in a limited colon environment, is an important research topic for colonoscopy image analysis systems [12,13,14,15,16].

The recent monocular depth estimation technology shows comparable performance to the conventional stereo depth estimation method [17]. In the study of colonoscopy depth estimation using a monocular supervised learning method [13,14,15], conditional random field, pix2pix [18], and a conditional generative adversarial network (GAN) [19] were used as the depth prediction network. In the study of measuring the coverage of colonoscopy based on a self-supervised learning [6], the view synthesis loss [20] and the prediction of the camera intrinsic matrix in the network [21] are applied. However, the depth obtained by the monocular learning-based method often flickers depending on the scale ambiguity and prediction per single frame [22]. In recent research, recurrent depth estimation using temporal information [23] and multi-view reconstruction using spatial information [24] were proposed for using spatiotemporal information.

**Figure 1:**
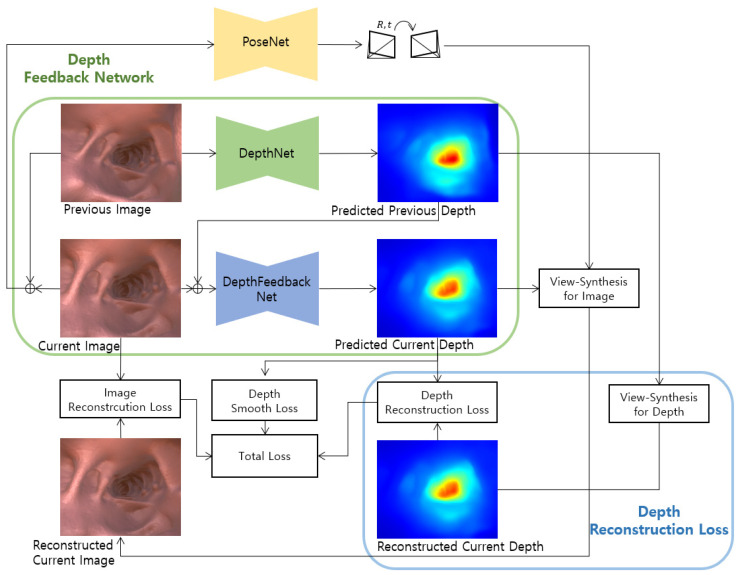
Our proposed self-supervised monocular network architecture. We introduce a depth feedback network and depth reconstruction loss.

It is our purpose for improving the existing self-supervised monocular depth estimation method through geometric consistency using a predicted depth. In this study, we propose a depth feedback network that inputs the predicted depth of the previous frame into the current frame depth prediction, and a depth reconstruction loss between the view synthesis of the predicted depth of the previous frame and the predicted depth of the current frame. Figure 1 shows the proposed FBNet structure including the depth feedback network and depth reconstruction loss.

The remainder of this paper is organized as follows. Section 2 presents recent research on colonoscopy depth estimation and unsupervised monocular depth estimation. Section 3 reviews the unsupervised monocular depth estimation used in this study and introduces the proposed depth feedback network and depth reconstruction loss. Section 4 performs a performance comparison with existing studies and proves the performance improvement for the network proposed by the ablation study. Finally, Section 5 presents the conclusion.

## 2. Related Works

The goal of this work is to improve the depth estimation performance of colonoscopy. The depth estimation study was mainly learned by a supervised method, but it is dependent on the image and depth pair data. However, the recent self-supervised method outperforms comparable performance to the supervised method. When it is difficult to obtain label data such as a colonoscopy image, the self-supervised method is more effective. In this work, the depth of colonoscopy is predicted by self-supervised learning. In addition, a monocular camera-based depth estimation technique is investigated according to the characteristics of colonoscopy. To this end, this section reviews the related work of colonoscopy depth estimation and unsupervised monocular depth and pose estimation.

### 2.1. Colonoscpy Depth Estimation 

The depth estimation network based on supervised learning is trained with data consisting of pairs of image and depth, like the autonomous driving dataset KITTI [25]. The KITTI dataset was acquired using multiple cameras and lidar sensors. However, it is a difficult problem to acquire actual depth data from colonoscopy images. Existing research creates a dataset from a CT-based 3D model to solve the scarce data. The 3D model is converted to an image dataset using 3D graphic engine software such as Blender or Unity. In the graphics engine, animation scenes are created by changing textures, creating virtual camera paths, and using various lights. The image and depth pairs to be used as the synthetic dataset are the outputs of each image and depth renderer in the produced animation scene [6,14].

Unlike the supervised method, which requires data consisting of pairs of image and depth, the unsupervised depth estimation network uses continuous colonoscopy images as training data. Therefore, the self-supervised method uses not only synthetic datasets, but also images taken from real patients or images from phantoms for network training [6,26]. 

As a colonoscopy study using depth estimation, Itoh et al. [5], Nadeem, and Kaufman [11] use depth estimation for polyp detection. In addition, Freedman et al. [6] and Ma et al. [27] apply dense 3D reconstruction to measure non-search areas of colonoscopy. In addition, there are adversarial training network-based approaches [12,14] that make composite images resemble real medical images, and unsupervised depth estimation studies to be applied to wireless endoscopic capsules [26].

### 2.2. Unsupervised Monocular Depth and Pose Estimation

A supervised learning method shows relatively good performance, but, in recent research, the unsupervised learning method also shows comparable performance [28]. Unsupervised learning is a suitable solution for the problem where it is difficult to acquire depth labels such as colonoscopy images. Garg et al. [29] propose a view synthesis that reconstructs the right image into the left image with the depth estimated from the left image in a pair of calibrated stereo images, and defines the difference between the reconstructed image from the right image and the left image as a reconstruction error. This has a problem in which a pre-calibrated pair must exist. Zhou et al. [20] propose a network that simultaneously estimates depth and ego-motion from a monocular sequence, and they apply view synthesis to reconstruct the image with the predicted pose and depth. They also use a mask that improves the explainability of the model. Godard et al. [30] applied a spatial transformer network (STN) [31], which is a completely differentiable sampling technique that does not need to simplify or approximate the cost function for the image reconstruction method. In addition, they proposed a photometric loss combining a structural similarity index measure (SSIM) [32] and L1 loss. Godard et al. [17] propose a minimum reprojection loss that uses a minimum value instead of an average in calculating the photometric error with adjacent images, reduces the artifacts of the image boundary, and improves the sharpness of the occlusion boundary. They also propose a multi-scale prediction to prevent the training target from being trapped in the local minimum with gradient locality by bilinear sampling. Recent approaches add loss [33], networks such as an optical flow network for motion information supplementation [34,35], and a feature-metric network for semantic information addition [36] and reduce the performance difference between monocular and stereo-based depth estimation.

However, this unsupervised learned depth is not guaranteed by a metric measure. That is, the network output is relative depth, and it is evaluated after scaling by the median value of the ground truth. Guizilini et al. [37] propose a velocity supervision loss based on the multiplication of the speed by the time between target and source frames for a scale-aware network.

Existing unsupervised learning models need to know the camera intrinsic matrix. Guizilini et al. [21] propose a network that can learn camera intrinsic parameters, and Vasiljevic et al. [38] propose a general geometric model [39] based on the neural ray surface that can learn depth and ego-motion without prior knowledge of the camera model.

## 3. Methods 

This section describes a self-supervised depth estimation network that estimates depth from adjacent input images. First, we review the main technologies of self-supervised learning based on previous studies. This review describes the notation and geometry model used in the proposed method. In this review, we also explain the loss to be used for the total loss. Then, the depth feedback network, depth reconstruction loss, and total loss proposed in this study are explained.

### 3.1. Self-Supervised Training

**Figure 2:**
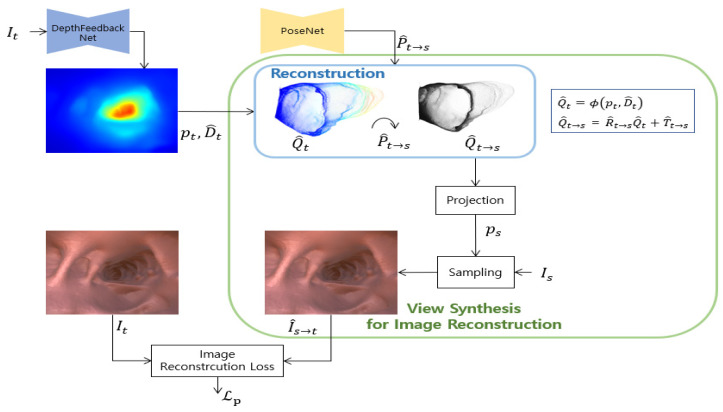
View synthesis structure for image reconstruction. This is a view synthesis process for self-supervised image reconstruction loss. The predicted depth ${\hat{D}}_{t}$ by the depth feedback network proposed in this work are reconstructed and transformed into a source viewpoint using predicted pose. ${\hat{I}}_{s\to t}$ is synthesized from $I_{s}$ by bilinear sampling using a pixel coordinate $p_{s}$ obtained by projecting reconstructed 3D points ${\hat{Q}}_{t\to s}$.

Following recent studies based on a self-supervised learning method [17,20], the depth network and the pose network are simultaneously learned. Networks are trained by minimizing the reconstruction error $L_{p}$ between the target image $I_{t}\;$and the image ${\hat{I}}_{s\to t}\;$reconstructed from the source image $I_{s}$ to the target view. Figure 2 shows this view synthesis process for self-supervised image reconstruction loss. 

First, pixel correspondence between the source image and the target image is required in the view synthesis process. This correspondence is used for sampling that transforms the source image into a target image. The pixel coordinate $p_{s}$ projected from the homogeneous pixel coordinate $p_{t}$ of the target image $I_{t}$ to the source image $I_{s}$ is shown below the equation using the predicted depth ${\hat{D}}_{t}$ and the predicted relative pose ${\hat{P}}_{t\to s}=\left({\hat{R}}_{t\to s},{\hat{T}}_{\;t\to s}\right)$.

(1) $$p_{s}=\;\pi \left({\hat{R}}_{t\to s}\phi \left(p_{t},{\hat{D}}_{t}\right)+{\hat{T}}_{t\to s}\right)$$

Here, π is a camera projection operation that converts the 3D point Q=(X,Y,Z) of the camera coordinate to the 2D pixel coordinate p=(u,v) of the image plane. ϕ is an unprojection that converts the homogeneous coordinates p and depth values d of the image into 3D points in the camera coordinate system, i.e.,

(2) $$\pi \left(Q\right)=\frac{1}{Z}KQ=\frac{1}{Z}\;\left[\begin{array}{ccc} f_{x} & 0 & c_{x}\\ 0 & f_{y} & c_{y}\\ 0 & 0 & 1 \end{array}\right]{\left[X\;Y\;Z\right]}^{T}$$

(3) $$\phi \left(p,d\right)=dK^{-1}p=d\;{\left[\begin{array}{ccc} f_{x} & 0 & c_{x}\\ 0 & f_{y} & c_{y}\\ 0 & 0 & 1 \end{array}\right]}^{-1}{\left[u\;v\;1\right]}^{T}$$

where K is the camera intrinsic matrix. $f_{x}$, $f_{y}$ are the focal length and $c_{x}$, $c_{y}$ represent the principal point. 

**Figure 3:**
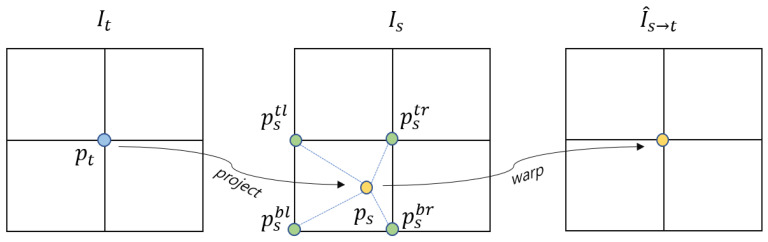
Bilinear sampling process. This is the process of projecting each point $p_{t}$ of target image $I_{t}$ to the source image $I_{s}$, and inputting a pixel value obtained by interpolating the surrounding pixels of the projected point into $p_{t}$ of ${\hat{I}}_{s\to t}$. As a result, the image ${\hat{I}}_{s\to t}$ at the viewpoint $I_{t}$ is synthesized from $I_{s}$.

To the next, the target image $I_{t}$ can be reconstructed from the source image $I_{s}$ by sampling the coordinates $p_{s}$ projected to the source image. Binary sampling is performed to calculate $I_{s}\left(p_{s}\right)$ in the discrete image space because $p_{s}$ is continuous. The discrete image ${\hat{I}}_{s\to t}\left(p_{t}\right)$ is obtained by transforming $I_{s}\left(p_{s}\right)\;$calculated as the neighboring pixel value of $I_{s}\left(p_{s}\right)$. The sampling can be formulated as:

(4) $${\hat{I}}_{s\to t}\left(p_{t}\right)=I_{s}\left(p_{s}\right)=\sum_{i\in \left\{t,b\right\},j\in \left\{r,l\right\}}w^{i,j}I_{s}\left(p_{s}^{i,j}\right)$$

where $p^{neighbor}\in \left\{p_{s}^{tl},p_{s}^{tr},p_{s}^{bl},p_{s}^{br}\right\}$ includes the values of the top-left, top-right, bottom-left, and bottom-right pixels of $p_{s}$, and $w^{i,j}$ is the weight value according to the distance between $p_{s}$ and $p^{neighbor}$, and $\underset{i,j}{\sum }w^{i,j}=1$. This bilinear sampling process is shown in Figure 3.

#### 3.1.1. Image Reconstruction Loss

Following Reference [30], the evaluation of the similarity in pixels between the target image $I_{t}$ and the reconstructed image ${\hat{I}}_{s\to t}$ from the source image can be formulated as follows by combining the SSIM and L1 distances.

(5) $$pl\left(I_{t},{\hat{I}}_{s\to t}\right)=\alpha \;\frac{\left(1-SSIM\left(I_{t},{\hat{I}}_{s\to t}\right)\right)}{2}+\left(1-\alpha \right){\left\|I_{t}-{\hat{I}}_{s\to t}\right\|}_{1}$$

where α=0.85 is a balancing weight and SSIM is a method of comparing and evaluating the quality of the predicted image with the original image. It is an index frequently used for depth estimation [17,21,23,33,37]. The SSIM between two images $I_{x}$ and $I_{y}$ is defined by:

(6) $$SSIM\left(I_{x},I_{y}\right)=\frac{\left(2\mu_{x}\mu_{y}+c_{1}\right)\left(2\delta_{xy}+c_{2}\right)}{\left(\mu_{x}^{2}+\mu_{y}^{2}+c_{1}\right)\left(\delta_{x}^{2}+\delta_{y}^{2}+c_{2}\right)}$$

where $\mu_{x},\;\mu_{y}$ are the average values, $\delta_{x}$, $\delta_{x}$ are the variances, $\delta_{xy}$ is the covariance of the two images, and $c_{1},c_{2}$ are stabilized variables.

The set of source images $S\in \left\{s_{1},\;s_{2},\;\ldots \right\}$ is composed of frames adjacent to the target image in self-supervised learning. The number of predicted target images ${\hat{I}}_{s\to t}$ varies depending on the number of image groups in the adjacent frame. The existence of the occluded area of the object according to the camera movement or the structure in the scene increases the photometric loss. As shown in Reference [17], the minimum photometric loss is adopted by applying the most consistent source image among the source image sets.

(7) $${ℒ}_{p}=\underset{S}{\min}pl\left(I_{t},{\hat{I}}_{s\to t}\right)$$

**Figure 4:**
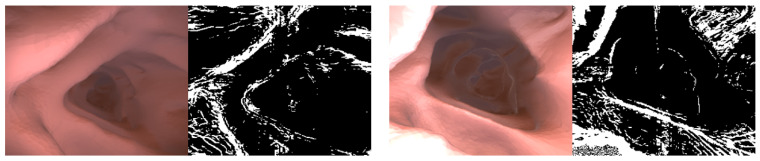
Auto-masking. Shows the auto-masking result learned in the experiment. Most of the colonoscopy images are flat areas and are calculated as black (μ=0) by auto-masking, and photometric loss is calculated based on the edge or textured area (μ=1).

Self-supervised learning works assuming a moving camera and a static scene. However, the dynamic camera movement, the object moving in the same direction as the camera, and the large texture-free area cause the problem of measuring infinite depth. The auto-masking technique introduced in Reference [17] is applied to the photometric loss to remove static pixels and reduce hole problems. Auto-masking for static pixel removal is set when the un-warped photometric loss $pl\left(I_{t},I_{s}\right)$ is greater than the warped photometric loss $pl\left(I_{t},{\hat{I}}_{s\to t}\right)$ and can be formulated as the following equation.

(8) $$\mu =\underset{S}{\min}pl\left(I_{t},{\hat{I}}_{s\to t}\right)<\;\underset{S}{\min}pl\left(I_{t},I_{s}\right)$$

where μ∈[0,1] is a binary mask, and the intermediate experimental result in which the texture-free area by auto-masking is removed is shown in Figure 4. The photometric loss value of the area erased by auto-masking is not used for network training. The result image below shows that the existing auto-masking works normally even in the colonoscopy image.

#### 3.1.2. Depth Smoothness Loss

Since the depth discontinuity depends on the gradients $\delta I_{t}$ of the image, the edge-aware term is used together as in previous studies [17,36,37] to limit the high depth gradient $\delta {\hat{D}}_{t}$ for the texture-less region.

(9) $${ℒ}_{s}\left({\hat{D}}_{t}\right)=\left|\delta_{x}{\hat{D}}_{t}\right|e^{-\left|\delta_{x}I_{t}\right|}+\left|\delta_{y}{\hat{D}}_{t}\right|e^{-\left|\delta_{y}I_{t}\right|}$$

#### 3.1.3. Multi-Scale Estimation

In the previous research [17], multi-scale depth prediction and reconstruction is performed to prevent falling into local minima by the bilinear sampler. Holes tend to occur at the predicted depth in the low-texture region of the low-resolution layer, and Reference [17] proposes to upscale the depth to the input image scale to reduce the occurrence of holes. This study also adopts the intermediated layer upscale based on multi-scale depth estimation, which upscales the intermediate resulting depth of each layer of the decoder to the resolution of the input image, reprojects, and resamples it.

For each layer, the photometric loss is calculated as an average, and the depth smooth loss is weighted according to the resolution size of each layer region, as shown in Reference [37]. Finally, the depth smoothness loss is formulated as follows.

(10) $${ℒ}_{s}\left({\hat{D}}_{t}\right)=\frac{1}{N}\;\underset{n}{\sum }\frac{{ℒ}_{s}\left({\hat{D}}_{t,n}\right)}{2^{n}}$$

where N is the number of intermediate layers of the backbone decoder, and n is the scale factor of the intermediate layer resolution divided by the input.

### 3.2. Improved Self-Supervised Training

As mentioned above, recent research studies use a method of adding a network reinforcing feature or segmentation information [36,40] and a loss model for geometry or light [16,33]. Intuitively, feature and semantic information are not appropriate for depth prediction due to the characteristics of colonoscopy images. Therefore, in this study, we add information about geometric consistency to the network and loss function.

In this work, in order to improve the performance of monocular depth estimation, we propose a depth reconstruction loss that compares the similarity between the warped previous depth and the current depth. We also propose a depth feedback network that inputs the previous depth into the current depth prediction network.

#### 3.2.1. Depth Reconstruction Loss

Image reconstruction loss is calculated as the similarity between the synthesized source image converted at the target viewpoint by sampling and the target image. Similarly, the synthesis depth converted from the source depth to the target viewpoint can be compared with the target depth. This limits the prediction range of depth due to the assumption that the depths of geometrically adjacent frames will be consistent. Similar to Reference [16], this work focuses on the similarity of predicted depth maps between adjacent frames.

Reference [16] uses the target view 3D points ${\hat{Q}}_{t}=\phi \left(p_{t},{\hat{D}}_{t}\right)\;$lifted from ${\hat{D}}_{t}$ and the transformed 3D points ${\hat{Q}}_{s\to t}$. Here, ${\hat{Q}}_{s\to t}={\hat{R}}_{s\to t}\;{\hat{Q}}_{s}+{\hat{T}}_{s\to t}$ is a 3D point obtained by converting the 3D point ${\hat{Q}}_{s}$ into a target image viewpoint with a predicted inverse pose ${\hat{P}}_{t\to s}^{-1}$. They use a loss that minimizes the error of the identity matrix and the transform matrix between 3D points ${\hat{Q}}_{s\to t}$ and ${\hat{Q}}_{t}$.

**Figure 5:**
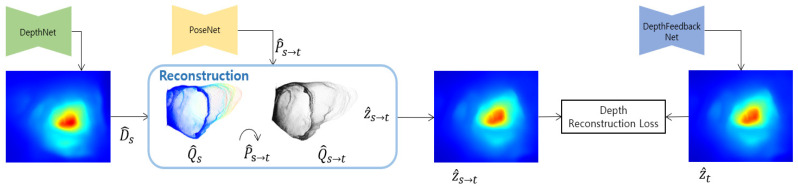
View synthesis structure for depth reconstruction. Similar to image reconstruction, the depth of source is reconstructed and transformed. ${\hat{z}}_{s\to t}$ is extracted from the reconstructed ${\hat{Q}}_{s\to t}$ for depth reconstruction loss. Finally, the loss between ${\hat{z}}_{s\to t}$ and ${\hat{z}}_{t}\left(={\hat{D}}_{t}\right)$ is calculated.

Similarly, this work minimizes the distance between depth maps. The depth scale of 3D points ${\hat{Q}}_{s\to t}=\left[{\hat{x}}_{s\to t},{\hat{y}}_{s\to t},{\hat{z}}_{s\to t}\right]$ and ${\hat{Q}}_{t}=\left[{\hat{x}}_{t},{\hat{y}}_{t},{\hat{z}}_{t}\right]$ may have different scales, according to the depth scale ambiguous problem of self-supervised monocular learning. We use force to maintain depth consistency in adjacent frames by adding a loss that minimizes the difference between reconstructed depth ${\hat{z}}_{s\to t}$ and predicted depth ${\hat{z}}_{t}$. Figure 5 shows the detailed structure diagram of view synthesis for depth reconstruction loss. Proposed depth reconstruction loss is formulated as follows by combining SSIM and L1 similarly to image reconstruction loss.

(11) $${ℒ}_{d}\left({\hat{z}}_{t},{\hat{z}}_{s\to t}\right)=\alpha \frac{\left(1-SSIM\left({\hat{z}}_{t},{\hat{z}}_{s\to t}\right)\right)}{2}+\left(1-\alpha \right){\left\|{\hat{z}}_{t}-{\hat{z}}_{s\to t}\right\|}_{1}$$

where a=0.15 is a balancing coefficient.

#### 3.2.2. Depth Feedback Network

Since the model trained by the general self-supervised monocular depth estimation method predicts the relative depth for a single frame, flicker may occur when applied to consecutive images [22]. Patil et al. [23] improves the depth accuracy based on spatiotemporal information by concatenating the encoding output of the previous frame with the encoding output of the current frame and decoding it. In a recent study [22], performance was improved by proposing optical flow-based loss including geometry consistency, but real-time execution is impossible because of an additional operation that requires learning at test time.

We propose a depth feedback network in which the depth network receives both the current image and the previous depth. This forces the network to extract the current depth based on the previous depth, as the network itself learns both the current image and the previous depth. We expect the accuracy improvement because the depth reconstruction loss and the depth feedback loss use spatiotemporal information of the depth of the adjacent frame.

The proposed depth feedback network consists of ${\hat{D}}_{s}=Net_{depth}\left(I_{s}\right)$ predicting the depth ${\hat{D}}_{s}$ of the source frame and ${\hat{D}}_{t}=Net_{DepthFeedback}\left(\left[I_{t},{\hat{D}}_{s}\;\right]\right)$ predicting the depth ${\hat{D}}_{t}$ of the target frame. Here, $\left[I_{t},{\hat{D}}_{s}\;\right]$ is the concatenation of $I_{t},{\hat{D}}_{s}$.

#### 3.2.3. Final Loss

All losses are summed according to scale N of multi-scale estimation. Final loss function is defined as:

(12) $$L=\underset{N}{\sum }\mu {ℒ}_{p}^{n}+\alpha {ℒ}_{s}^{n}+\beta {ℒ}_{d}^{n}$$

Here, α, β are the scale correction values for each loss, and we set α=0.001, β=0.05.

## 4. Experiments

### 4.1. Experimental Setup

The hardware environment used in our training and testing experiments is a desktop with Intel(R) i9-10900KF CPU 3.7GHz of Intel, 32G DDR4 memory of Samsung and GeForce RTX 3090 24G of Nvidia. The software environment was tested on the deep learning platforms pytorch, CUDA-10.1, and cudnn-7 on the operating system Ubuntu 18.04 LTS.

The proposed depth feedback network and depth reconstruction network test the Packnet-SfM [37] model as a baseline. The depth and pose network are trained 30 epoch learning, a batch size of 8, an initial depth, a pose learning rate of $2\cdot 10^{-4}$, and an input resolution of 256 × 256. The target frame is set as the current frame and the source frame is set as the previous frame. Unwritten parameters followed the values of Packnet-SfM.

The camera intrinsic matrix K must be known to train view synthesis based on monocular depth estimation. A recent work [21] proposed a model that can train a camera intrinsic matrix at training time. In this experiment, the above model is trained using the dataset to be used in our experiment, and the output camera intrinsic matrix K value of the above model is used as all K values in our experiment. In the above model training, the translation loss was excluded, as mentioned in their paper, as ineffective.

#### 4.1.1. Datasets

Image and depth pair images are used to evaluate the performance of depth estimation. However, it is difficult to measure the depth of colonoscopy with a sensor, such as lidar, to obtain the actual depth label. Therefore, synthetic datasets that extract images and depth from 3D modeling data are used for evaluation in the field of colonoscopy depth estimation.

To the best of our knowledge, a publicly available synthetic colonoscopy image and depth dataset is the University College London (UCL) dataset [14]. They created a 3D model from human colonography scan images, and they obtained about 16,000 images and depth maps by moving virtual cameras and lights along the path of the colon using the game engine Unity. In the case of Reference [6], 187,000 images and depth maps of synthetic datasets were obtained in a similar way, but only the synthetic images were released. The UCL dataset used for evaluation is divided into training and test datasets at a ratio of 6:4 similar to the previous unsupervised learning study [6]. In addition, 3D reconstruction is performed on the image sequence taken from Koken’s LM-044B colonoscopy simulator.

#### 4.1.2. Evaluation Metrics

The four error metrics, absolute relative error (AbsRel), square relative error (SqRel), root mean squared error (RMSE), and RMSE(log) used in recent related studies [17,20,37] are used for quantitative evaluation of the self-supervised monocular depth estimation proposed in this work. Additionally, the threshold accuracy (δ) metric is used to evaluate the accuracy. The error metric and accuracy metric are formulated as follows.

(13) $$AbsRel=\frac{1}{N}\overset{N}{\underset{i}{\sum }}\frac{\left|D_{i}^{GT}-{\hat{D}}_{i}\right|}{D_{i}^{GT}}$$

(14) $$SqRel=\frac{1}{N}\overset{N}{\underset{i}{\sum }}\frac{{\left|D_{i}^{GT}-{\hat{D}}_{i}\right|}^{2}}{D_{i}^{GT}}$$

(15) $$RMSE=\sqrt{\frac{1}{N}\overset{N}{\underset{i}{\sum }}{\left|D_{i}^{GT}-{\hat{D}}_{i}\right|}^{2}}$$

(16) $$RMSE\left(\log\right)=\sqrt{\frac{1}{N}\overset{N}{\underset{i}{\sum }}{\left|logD_{i}^{GT}-log{\hat{D}}_{i}\right|}^{2}}$$

(17) $$Threshold\;accuracy\left(\delta <thr\right)=max\left(\frac{D_{i}^{GT}}{{\hat{D}}_{i}}\;,\;\frac{{\hat{D}}_{i}}{D_{i}^{GT}}\;\right)$$

Here, $D_{i}^{GT}$ and ${\hat{D}}_{i}$ are values of the ground truth depth and predicted depth corresponding to pixel i, respectively, and N is the total number of pixels. thr uses $\left(1.25,\;1.25^{2},1.25^{3}\right)$ as in previous studies.

### 4.2. Comparison Study

A comparison study is performed to evaluate the performance of the proposed algorithm. There are [6,14] papers that have previously been evaluated with the UCL dataset. Reference [14] was performed and tested based on extended pix2pix, which is a supervised learning method, and Reference [6] was performed using self-supervised learning. These results are cited in their paper, and we note that the detailed composition may differ from our evaluation datasets because we divide the datasets in sequence units for learning adjacent images.

In the comparative experiment, we compare the performance while changing the backbone of the depth network of Monodepth2 [17], Packnet-SfM [37], and FBNet to Resnet18, Resnet50 [41], and Packnet [37]. All pose networks used Resnet18 as the backbone, and the number of 3D convolutional filters of the backbone network Packnet was set to 8. 

First, Table 1 shows the results of quantitative performance evaluation based on evaluation metrics. The quantitative performance of the proposed network shows higher performance in most items than other control group networks. FBNet using Resnet50 shows the highest performance in threshold accuracy, and FBNet using Packnet shows the highest performance in an absolute relative error.

**Figure 6:**
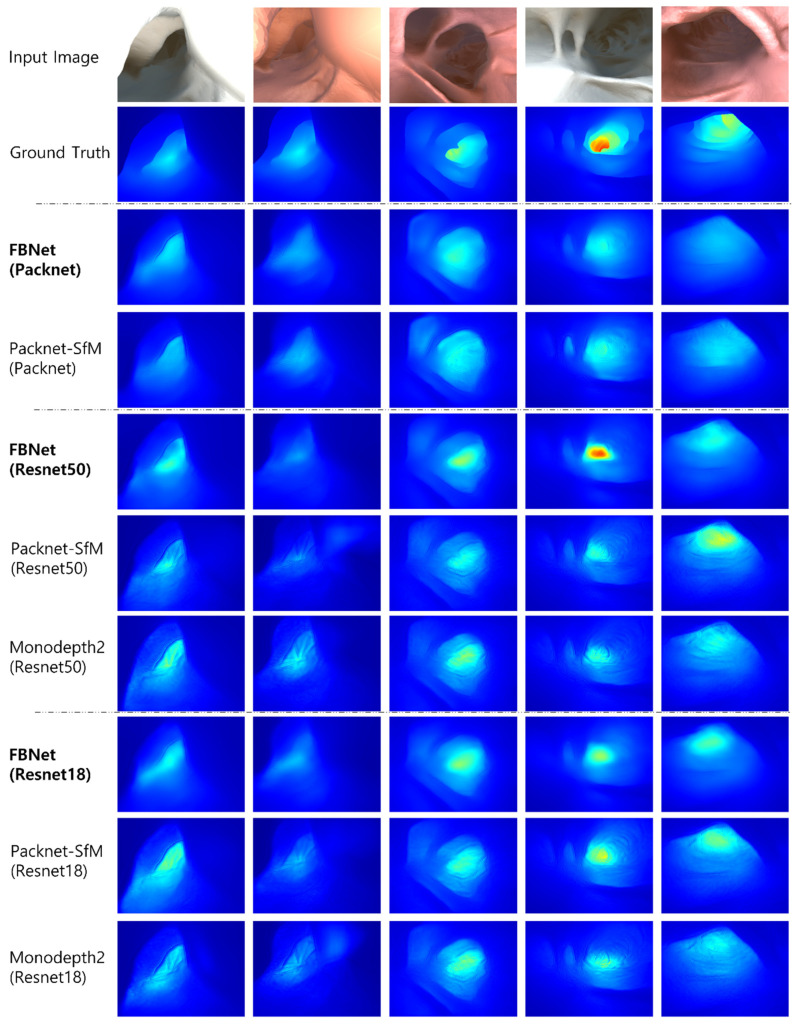
Qualitative results for depth estimation. Compared to other methods, FBNet has less noise due to texture. This is because geometry consistency information using a depth feedback network and depth reconstruction loss were used.

Next, the input image, ground truth depth, and qualitative comparison image of UCL Datasets are shown in Figure 6. In the evaluation, the median value of predicted depth is scaled by a median value of ground truth depth. The predicted depth is displayed in color from blue to red, from the nearest to the farthest. Each column is the output of the predicted depth from the input image for each network. In the qualitative performance evaluation, the phenomenon in which the shape of the image texture is propagated to the predicted depth has been reduced. It also can be seen that FBNet(Resnet50) predicts a deep depth that is not predicted by other networks. 

**Figure 7:**
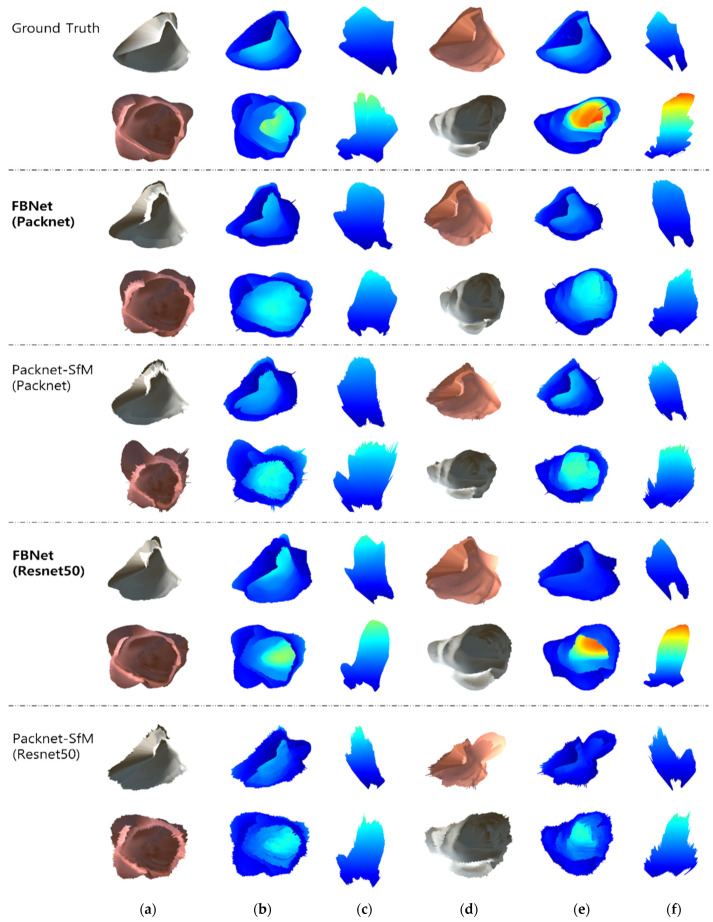
Qualitative results for 3D reconstruction. We compare the results of 3D reconstruction of the images in the first to fourth columns of Figure 6. (**a**,**d**) are the results of 3D reconstruction image mapping. (**b**,**e**) are expressed as colormaps according to the depths of (**a**,**d**). (**c**,**f**) are the top-view of (**b**,**e**).

In addition, 3D reconstruction is performed by un-projection based on the predicted depth and intrinsic camera matrix. Figure 7 shows the qualitative evaluation of 3D reconstruction results of FBNet and Packet-SfM. In addition, the backbone of each depth network is tested on Packnet and Resnet50. The result is shown the front view captured from the position of the predicted camera pose and the top view taken from the top by moving the virtual camera. The mapped depth image is the result of Figure 6. Compared to Packnet-SfM, the proposed FBNet shows robustness against noise caused by texture. This is an improvement in qualitative performance as FBNet applies geometric consistency using depth of adjacent frames.

**Figure 8:**
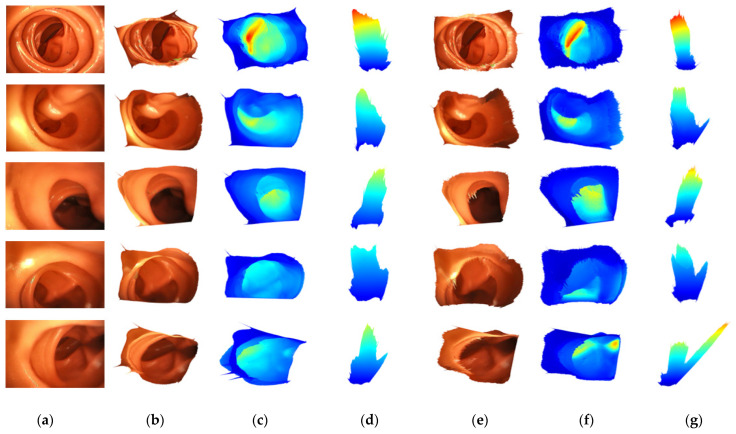
Qualitative results for 3D reconstruction. (**a**) is an input image taken with the camera in colonoscopy simulation. (**b**–**d**) are results of FBNet. (**e**–**g**) are results of Packnet-SfM. (**b**,**e**) are the results of 3D reconstruction image mapping. (**c**,**f**) are expressed as colormaps according to the depths of (**b**,**e**). (**d**,**g**) are the top-view of (**c**,**f**).

Finally, Figure 8 shows a 3D reconstruction comparison experiment for the image captured by the colonoscopy simulator. The reconstruction result is shown in the same way as in the above experiment. Only the input images are different. Since the captured image has no ground truth, it is scaled by multiplying it by a constant value. There was a noise for light reflection that could not be observed in UCL datasets, and the proposed FBNet is more robust to lighting noise than Packnet-SfM.

### 4.3. Ablation Study

The evaluation of the performance improvement due to the depth feedback network and depth reconstruction loss proposed by FBNet is performed as an ablation study and is shown in Table 2. In this experiment, we remove the proposed factor and confirm the increased performance as compared to the baseline model.

Table 2 shows that the performance improvement by the depth feedback network is higher than that of the depth reconstruction loss. In addition, it was confirmed that the performance of Packnet was better than Resnet50 in the KITTI dataset [37], while the accuracy and error metric of the two backbones in the UCL dataset was almost similar in both the baseline and FBNet models. This seems to mean that, in the case of colonoscopy images, the effect of the deep-layer network is not large because the features are lacking and there are many texture-less areas.

Compared to the baseline model, FBNet uses one more depth feedback network, so it has more training parameters. In the inference time, the depth is predicted with the depth network only in the first frame, and the depth feedback network is used in the subsequent frames. Therefore, the computational load that increases in actual running time is an operation according to the depth input channel insertion.

## 5. Discussion

In this study, a general self-supervised monocular depth estimation methodology is used for depth estimation of colonoscopy images. The existing depth estimation research was conducted based on the autonomous driving datasets KITTI. This dataset can get geometric information from enough texture of the image, but, in the case of colonoscopy images, almost all areas are texture-less. In this study, we propose the FBNet that applies both depth feedback network and depth reconstruction loss to increase geometry information. 

The proposed FBNet was evaluated quantitatively and qualitatively using images taken from a colonoscopy simulator and UCL datasets. We confirmed the lower error metric and higher accuracy metric. In addition, through qualitative evaluation, it was confirmed that it is robust to depth noise and specular reflection noise.

Our future research will focus on the colonoscopy map and path generation for autonomous robotic endoscopes. The proposed depth estimation network will continue to be used for solving a scale-ambiguity problem, image registration for simultaneous localization and mapping (SLAM), and path planning. In addition, the current method has limitations in that each model must be trained according to the colonoscopy device. In order to apply to more general devices, we will apply a method of estimating camera parameter values to the model.

## Figures and Tables

**Table 1:** Quantitative performance comparison of the proposed algorithm on the UCL datasets. In the learning column, S refers supervised learning and SS refers self-supervised learning. For Abs Rel, Sq Rel, RMSE, and RMSElog lower is better, δ < 1.25, $\delta \;<\;1.25^{2}$, $\delta \;<\;1.25^{3}$ higher is better. The best performance of the test for each backbone is indicated in bold, and the best performance of all experiments is indicated by an underline.

Learning	Method	Backbone	Abs Rel	Sq Rel	RMSE	RMSElog	δ < 1.25	$\delta \;<\;1.25^{2}$	$\delta \;<\;1.25^{3}$
S	Rau [14]		0.054	-	-	-	-	-	-
SS	Freedman [6]	Resnet18	0.168	-	-	-	-	-	-
	Monodepth2 [17]	Resnet18	0.163	2.157	10.134	0.211	0.784	0.941	0.979
	Packnet-SfM [37]	Resnet18	0.121	1.150	7.957	0.165	0.868	0.966	0.988
	FBNet	Resnet18	0.108	1.060	7.369	0.149	0.904	0.974	0.991
	Monodepth2	Resnet50	0.123	1.357	7.710	0.157	0.880	0.969	0.989
	Packnet-SfM	Resnet50	0.115	1.086	7.570	0.160	0.886	0.971	0.989
	FBNet	Resnet50	0.098	0.751	6.432	0.134	0.919	0.981	0.993
	Packnet-SfM	Packnet	0.116	1.091	7.806	0.159	0.884	0.971	0.990
	FBNet	Packnet	0.096	0.843	7.147	0.139	0.912	0.977	0.992

**Table 2:** Ablation study on the FBNet. We perform the ablation study under the same conditions as the comparative experiment. Performance is shown when depth reconstruction loss and depth feedback network are removed from the proposed full network.

Method	Backbone	Abs Rel	Sq Rel	RMSE	RMSElog	δ<1.25	$\delta <1.25^{2}$	$\delta <1.25^{3}$
FBNet	Resnet50	0.098	0.751	6.432	0.134	0.919	0.981	0.993
FBNet w/o Depth Reconstruction Loss		0.102	0.875	7.093	0.147	0.908	0.978	0.992
FBNet w/o Depth Feedback Network		0.107	0.824	6.453	0.146	0.906	0.973	0.989
Baseline		0.115	1.086	7.57	0.16	0.886	0.971	0.989
FBNet	Packnet	0.096	0.843	7.147	0.139	0.912	0.977	0.992
FBNet w/o Depth Reconstruction Loss		0.1	0.846	7.144	0.143	0.909	0.978	0.992
FBNet w/o Depth Feedback Network		0.106	1.029	7.941	0.146	0.894	0.975	0.992
Baseline		0.116	1.091	7.806	0.159	0.884	0.971	0.99
